# SARS-CoV-2 Infection and Adverse Maternal and Perinatal Outcomes: Time-to-Event Analysis of a Hospital-Based Cohort Study of Pregnant Women in Rio de Janeiro, Brazil

**DOI:** 10.3390/v17020207

**Published:** 2025-01-31

**Authors:** Michelle Brendolin, Mayumi Duarte Wakimoto, Raquel de Vasconcellos Carvalhaes de Oliveira, Larissa Rangel Mageste, Karin Nielsen-Saines, Patricia Brasil

**Affiliations:** 1 Maternity Department, Adão Pereira Nunes Hospital, Duque de Caxias 25.211-970, Brazil; mobrendolin@gmail.com; 2 Instituto Nacional de Infectologia Evandro Chagas, Fiocruz, Rio de Janeiro 21.040-360, Brazil; mayumi.wakimoto@ini.fiocruz.br (M.D.W.); raquel.vasconcellos@ini.fiocruz.br (R.d.V.C.d.O.); larissarangel05@gmail.com (L.R.M.); 3 Pediatric Infectious Diseases Division, David Geffen School of Medicine, University of California, Los Angeles, CA 90024, USA; knielsen@mednet.ucla.edu

**Keywords:** SARS-CoV-2, COVID-19, pregnancy, perinatal outcomes, mortality, preterm birth, death maternal, adverse outcomes

## Abstract

Understanding perinatal health outcomes following SARS-CoV-2 infection during pregnancy necessitates large-scale studies of mother-infant dyads. Hospital-based studies of pregnant women and their neonates provide valuable insights within the field of perinatal health research. The aim of this study was to evaluate the effect of SARS-CoV-2 infection on maternal and perinatal outcomes among hospitalized pregnant women in Rio de Janeiro during the COVID-19 pandemic. Methods: The study consisted of a time-to-event analysis of a hospital-based cohort of 1185 pregnant women ≥ 16 years and their infants from May 2020 to March 2022. Pregnant women were classified as infected if they had a SARS CoV-2 positive RT-PCR or a positive rapid antigen test. An exploratory analysis of qualitative variables was conducted with calculation of absolute and relative frequencies and calculation of 95% confidence intervals. Survival functions were estimated by the Kaplan–Meier method, and the Cox proportional hazards model was employed to interpret the effects of SARS-CoV-2 infection on time to adverse maternal and perinatal outcomes, adjusted for vaccination, comorbidity, and gestational trimester. Results: A total of 21% (249/1185) women were infected with SARS-CoV-2, with a median age of 26 (range: 16–47). Cesarean section deliveries were performed in 57% (135/237) SARS CoV-2+ participants vs. 43% (391/914) of uninfected participants, *p* < 0.001. Intensive care unit admission and/or death occurred in 68 of 1185 participants (5.7%), 44 of 249 participants (17.7%) infected with SARS CoV-2 vs. 24 of 936 uninfected participants (2.5%). All 21 participants who died were unvaccinated against COVID-19. Women infected with SARS-CoV-2 were at greater risk of adverse maternal outcomes (crude HR: 5.93, 95% CI: 3.58–9.84; adjusted HR: 5.47, 95% CI: 3.16–9.48) than uninfected pregnant women. SARS CoV-2 vertical transmission was observed in 6 of 169 (3.6%) tested neonates. Preterm deliveries occurred more frequently in patients testing positive for SARS-CoV-2 (30.7% vs. 23.6). In the survival analysis, no effect of SARS-CoV-2 infection was observed on prematurity (HR: 0.92, 95% CI: 0.68–1.23) and adverse perinatal outcomes, including fetal distress (HR: 1.29, 95% CI: 0.82–2.05), stillbirth (HR: 1.07, 95% CI: 0.48–2.38), and neonatal death (HR: 0.96, 95% CI: 0.35–2.67), even after adjusting for vaccination, comorbidity, gestational trimester, and periods of time. Conclusion: The risk of maternal death due to COVID-19 highlights the need for adequate preventive measures, particularly vaccination, during the prenatal and postpartum periods.

## 1. Introduction

The SARS-CoV-2 pandemic has led to a substantial global burden of disease, characterized by high rates of morbidity and mortality. A concerning trend observed during the pandemic was a significant increase in maternal mortality rates attributed to COVID-19 [1,2,3], with Brazil contributing for an important portion of global cases [4]. However, research on maternal and perinatal outcomes associated with COVID-19 in the Brazilian obstetric population remains limited [5,6]. In August 2020, the Pan American Health Organization (PAHO/WHO) issued an alert to member countries regarding the risks of COVID-19 in pregnancy and the vulnerability of pregnant women (PW), guaranteeing the continuity of prenatal care and attention to severe signs and symptoms during pregnancy [7].

Higher vulnerability of pregnant women can be explained by changes in the immune system or other physiological changes during pregnancy, such as increased heart rate, stroke volume, and oxygen consumption and decreased lung capacity. These factors may contribute to a greater risk of serious illnesses and increase the risk of venous thromboembolism four to fivefold [8,9,10]. Furthermore, clinical and social risk factors, high birth rates, and limited healthcare resources are also associated with adverse outcomes among pregnant women with severe acute respiratory syndrome (SARS) due to COVID-19 in low- and middle-income countries (LMICs) [11,12,13].

Despite vaccination being a crucial tool for controlling the pandemic, available since early 2021 in Rio de Janeiro, only 53.0% of pregnant women had received their first dose of the COVID-19 vaccine by July 2022 [14,15,16]. This low adherence was not unique to Brazil, as studies in the United States [17] showed similar trends in the first years of the pandemic. COVID-19 vaccination during pregnancy was often not recommended during prenatal care.

Prospective studies, while less common, offer more robust data for delineating the spectrum of poor obstetrical and perinatal outcomes. Since the time to event associated with severe maternal and perinatal COVID-19 cases is not yet fully explored, we used survival analysis as a powerful tool for investigating the relationship between the time since infection and clinical outcomes in diseases like COVID-19. We aimed to evaluate the effect of SARS-CoV-2 infection on maternal and perinatal outcomes in a reference maternity hospital, located in the city of Duque de Caxias, RJ, a low income setting for referrals of pregnant patients during the COVID-19 pandemic, while controlling for other relevant clinical factors. We also described the characteristics of maternal death that occurred within the cohort. This study employed an open cohort design, where women entered the study at different times. This approach enabled a more accurate assessment of the risk of these outcomes over time.

## 2. Materials and Methods

### 2.1. Study Population

Our prospective hospital-based cohort study was conducted in the maternity ward of a public hospital, in the city of Duque de Caxias, RJ, which is a reference hospital for medium- and high-risk pregnancies in a low-income setting. The city has a population of 1,729,360 inhabitants, with a gross domestic product per capita of $11,270 and a human development index (HDI) of 0.711, ranking 14th in the State of Rio de Janeiro [18,19].

**Figure 1:**
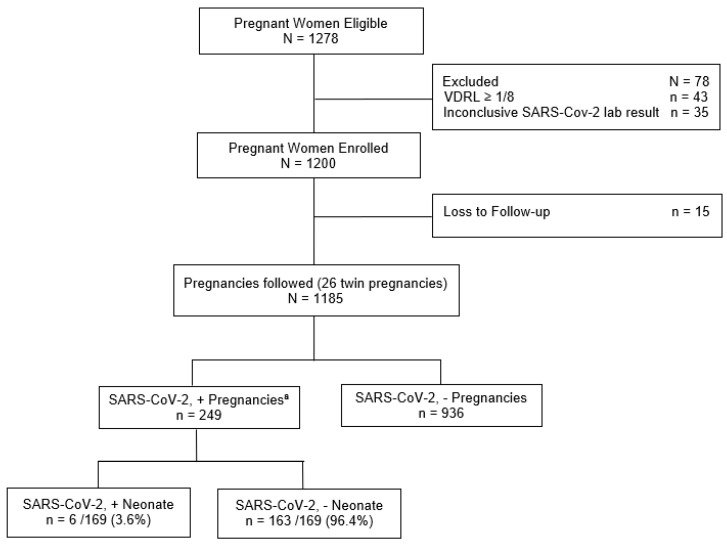
Flowchart of the pregnancies cohort in a high-risk maternity hospital, Rio de Janeiro, RJ, Brazil, 2020–2022. ^a^ SARS-CoV-2 + pregnancies: positive results, by reverse transcription polymerase chain reaction (RT-PCR) or COVID-19 antigen rapid test (RT); neonate positive results, by RT-PCR or IgA.

We enrolled all pregnant women aged ≥ 16 years, admitted for delivery or because of clinical or obstetric complications, from May 2020 to March 2022, who were accepted to do the SARS-COV-2 nasopharyngeal test. Follow-up was conducted during hospitalization and extended to puerperium (72 days postpartum made by phone calls and messages via WhatsApp). Pregnant women with syphilis (VDRL ≥ 1:8) or lost to follow-up and those with inconclusive RT-PCR results were excluded (Figure 1).

SARS-CoV-2 infection status was determined at study enrollment during hospitalization. The exposed group consisted of PW who were tested for SARS-CoV-2 (positive or negative). We used the COVID-19 rapid antigen test or real-time reverse transcription-polymerase chain reaction (RT-PCR) for viral genome detection and classified the PW as infected or uninfected based on these results. Exposed neonates were screened for SARS-CoV-2 by RT-PCR of nasopharyngeal swabs and measurement of anti-S IgM, IgA, and IgG antibodies in serum (3 mL) up to the third day of life. Neonatal infection was defined by viral RNA detection or IgM or IgA presence (available for 169 of 257 exposed neonates). Missing data were excluded from the analysis. Positivity for IgG was considered a marker of maternal passive immunity. To minimize the loss to follow-up of PW who were discharged from the hospital before delivery, all PW were contacted by telephone and WhatsApp to obtain information on gestational and perinatal outcomes. Additionally, at the end of the segment, a search was carried out using the National Immunization Program to verify the doses of the COVID-19 vaccine administered during pregnancy.

### 2.2. Variables

The study variables related to the sociodemographic and clinical characteristics of participants were as follows: age group (16–19 years, 20–34 years, 35–47 years), education (complete or incomplete primary education, complete or incomplete secondary education, and complete or incomplete higher education); color/race (other, black or multiracial, and white); marital status (married or living together, single, or separated); symptoms of COVID-19 (reported fever, cough, dyspnea, or myalgia) in the two groups (infected and not infected with SARS-CoV-2); COVID-19 vaccination (complete: two doses, ≥ 14 days before infection; incomplete: one dose, ≥ 14 days, before infection; unvaccinated: no vaccination or one dose of vaccine ≤ 14 days before diagnosis). The number of prenatal consultations was defined as “adequate” (six consultations or more) and “inadequate” (less than six consultations) during pregnancy; gestational trimester was defined as first trimester (up to 13 weeks); second trimester (14–27 weeks); and third trimester (28–42 weeks). Comorbidity was defined as the presence of any of the following: anemia; asthma; systemic arterial hypertension; diabetes mellitus; HIV; obesity. The age variable, in years, was verified by median and interquartile range. All recorded information was verified through interviews, filling out a collection instrument, and consulting the medical records of PW and pediatric patients.

Complications of pregnancy were defined as gestational hypertension (identification of arterial hypertension during the second half of pregnancy, in a previously normotensive pregnant woman with systolic blood pressure ≥ 140 mmHg and/or diastolic blood pressure ≥ 90 mmHg), pre-eclampsia (arterial hypertension, in a previously normotensive pregnant woman, from the 20th week of gestation, associated with significant proteinuria (≥ 300 mg/dL)), and gestational diabetes (any degree of glucose intolerance, with onset or first recognition during pregnancy).

The study’s outcomes of interest were (1) adverse maternal outcomes defined as admission to the intensive care unit (ICU) or maternal death (death during pregnancy or up to 72 days post-delivery); (2) four adverse perinatal outcomes: neonatal death (death of the newborn within 28 days of birth; stillbirth (fetal death before complete expulsion or extraction from the mother after 20 weeks of pregnancy); fetal distress (changes in fetal heart rate and cardiotocographic tracing); and prematurity (birth at less than 37 weeks of gestation).

The distribution of the following variables between pregnant women infected and not infected with SARS-CoV-2 was evaluated: sociodemographic variables, vaccination, prenatal consultation, gestational trimester, symptoms suggestive of COVID-19 (documented or tactile fever, chills, sore throat, headache, cough, runny nose, olfactory disorders, or taste disorders), comorbidities, complications during pregnancy, mode of delivery, and maternal transmission of SARS-CoV-2.

The predictors were vaccination (complete vs. incomplete/unvaccinated), comorbidity (yes or no), trimester, and period of time (up to June 2021, July–November 2021, and December 2021 +). The period of time was stratified according to the predominant viral lineage of COVID-19 variants: 2020–June 2021 (wild type and Gamma), July 2021 (Delta), and December 2021+ (Omicron) [20].

Positive test rates were calculated using the number of positive test results divided by the total number of tests conducted.

### 2.3. Statistical Analysis

We performed a descriptive analysis comparing the sociodemographic, gestational, and clinical characteristics of pregnant women with and without SARS-CoV-2 infection. The variables analyzed included age, race/ethnicity, education level, COVID-19 symptoms, vaccination status, comorbidities, gestational complications, number of prenatal visits, gestational trimester, type of delivery, and perinatal outcomes. Absolute and relative frequencies were used to describe the categories of each variable. Age, in years, was also assessed using the median with interquartile ranges (IQRs). Pearson chi-squared values were used to compare proportions, as well as their 95% confidence intervals (CI). A *p*-value < 0.05 was considered significant. To perform the exploratory analysis of time to adverse maternal and perinatal outcomes, the Kaplan–Meier method was performed (Supplementary Material Figure S1). Time to adverse maternal outcome, in days, was calculated from the date of first hospitalization to the date of maternal death or ICU admission. Pregnant women who did not die or who were admitted to the ICU by the final follow-up date were censored on the date corresponding to the day of hospital discharge. The time to adverse perinatal outcome, in days, was calculated on the date of birth outcome. Pregnant women with normal perinatal outcomes were censored on the date of the birth outcome. In cases with 0 days (<24h) from date of first hospitalization and the event (death), ICU admission, or hospital discharge, we considered the time as 1 day.

The semiparametric Cox model was used to interpret associations between SARS-CoV-2 infection and time to adverse maternal or perinatal outcomes. The subdistribution hazard model addressed competing risks (all adverse perinatal, neonatal death, stillbirth, fetal distress, and prematurity). For multiple covariate model analysis, we adjusted the effect of SARS-Cov-2 for vaccination (complete vs. incomplete/unvaccinated), presence of comorbidity, gestational trimester, and period of time (Supplementary Material Figure S2). These variables were selected according to potential relevance [20,21,22]. We considered the SARS-Cov-2 as an exposure variable and the others as confounders. Unadjusted estimates were called crude HR and confounder-adjusted estimates were called adjusted HR. The effects were interpreted by the hazard ratio (HR) in the simple (crude HR) and multiple (adjusted HR) models and their respective 95% confidence intervals (CI). We analyzed the Schoenfeld residual tests, and they did not indicate to reject the hypothesis of proportionality for all variables, except trimester. To address this, we considered the variable “trimester” as a strata variable in a stratified Cox model.

We present summary measures over time as Supplementary Material Table S1. The R package version 4.1 was used in the analysis [23]. The competing events analysis was conducted using the *mstate package* in the R software [24].

### 2.4. Sensitivity Analysis

Sensitivity analyses were conducted to determine whether the results changed when we omitted the following: (1) multiparous pregnant women, (2) pregnant women with preeclampsia and obesity, (3) pregnant women, and (4) newborns with follow-up time = 0 (Supplementary Material Table S2).

## 3. Results

Between May 2020 and March 2022, 1278 eligible PW were examined. From these, 1243 PW were enrolled in the study, from which 43 PW were excluded due to VDRL levels greater than or equal to 1/8 and 35 PW were excluded because of inconclusive SARS-CoV-2 laboratory results. From the resulting 1200 PW, we had 15 losses to follow-up. In total, 1185 pregnant participants were analyzed, from which 249 (21.0%) had laboratory-confirmed SARS-CoV-2 infection. A total of 1211 infants (26 twins) were delivered, 954 to 936 uninfected mothers and 257 to 249 mothers who tested positive for SARS CoV-2. Six of 169 infants born to SARS CoV-2-positive mothers who were tested for the virus (3.6%) had a laboratory confirmed diagnosis as shown (Figure 1).

Clinical and sociodemographic data of study participants are presented in Table 1. In summary, the median age was 26 years, ranging from 16–47 years. Of the 1185 participating PW, the average time between the start of monitoring and delivery was 12 days (2–247). The majority of participants were between the ages of 20 and 34; of other races/ethnicities; and with complete/incomplete high school education and adequate prenatal care. The percentage of infected women having complete (twodoses) vaccination (21.8%) was higher than that of the uninfected women (16.6%). However, the overall vaccination rate was lower among infected participants (69.8%) than among uninfected participants (66.1%), representing a difference of 3.7%. Among the 249 infected pregnant participants, 138 (55.4%) were symptomatic. The most common symptoms were cough (41.8%), fever (39%), and dyspnea (22.1%), as seen in Table 1.

High blood pressure and anemia were the most frequent chronic conditions, while pre-eclampsia and gestational diabetes were the most frequent gestational complications observed in pregnant patients in the cohort. Cesarean sections accounted for 45.7% of births, with a higher proportion among PW infected with SARS-CoV-2 (57.0%), as shown in Table 1.

### 3.1. SARS-CoV-2 and Maternal Outcomes

**Figure 2:**
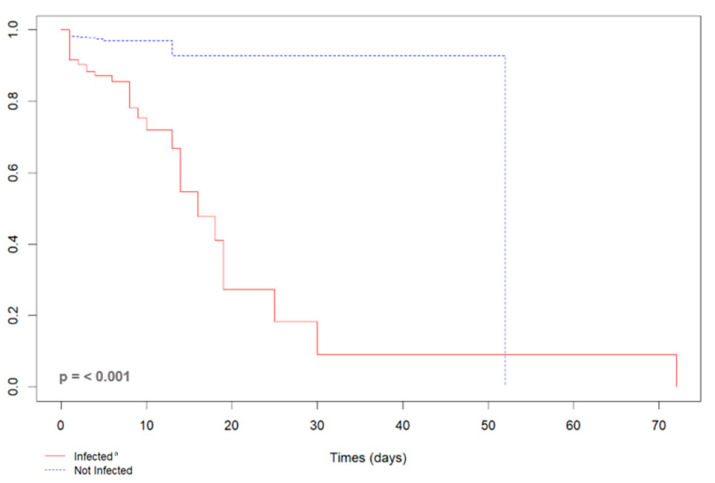
Kaplan–Meier curve of the length of stay of 72 pregnant women due to a serious maternal outcome (maternal death or admission to the ICU) according to the SARS-CoV-2 infection status, Rio de Janeiro, Brazil, 2020–2022. ª Infected by SARS-CoV-2, RT-PCR or rapid COVID-19 test positive; not infected with SARS-CoV-2, negative RT-PCR or rapid COVID-19 test.

In total, 5.7% (*n* = 68) of 1185 pregnant participants recruited to the cohort had a serious maternal outcome, with 64 ICU admissions and 21 maternal deaths. The majority of adverse outcomes, 64.7% (*n* = 44), occurred in participants infected with SARS-CoV-2. Among 249 infected participants, 42.4% had an adverse outcome, versus 313 of 936 uninfected participants (33.4%; *p* = 0.004). The indication for cesarean section was severity of the disease in 58.8% (*n* = 40). The Kaplan–Meier survival curve indicated that the time to development of an adverse maternal outcome (maternal death or admission to the ICU) was shorter in pregnant participants infected with SARS-CoV-2 as compared to uninfected participants. The risk of serious maternal outcomes within 16 days was significantly higher in those participants infected with SARS-CoV-2, as shown in Figure 2.

Pregnant participants with SARS-CoV-2 had a higher risk of experiencing an adverse maternal outcome (crude HR: 5.93, 95% CI: 3.58–9.84; adjusted HR: 5.47, 95% CI: 3.16–9.48; all Schoenfeld residuals were greater than 0.05) than uninfected pregnant participants. In the survival analysis, adverse maternal outcome and SARS-CoV-2 infection were categorized as symptomatic (*n* = 138; 53.7%) and asymptomatic (*n* = 119; 46.3%). Pregnant participants with symptomatic SARS-CoV-2 infection had a higher risk of serious maternal outcomes (crude HR: 3.90, 95% CI: 3.02–5.02; adjusted HR: 3.90, 95% CI: 3.02–5.02, *p*-value = < 0.001).

Of the 21 maternal deaths, 81% (17/21) were associated with COVID-19 (representing a fatality rate of 6.8%): 11 participants had severe acute respiratory syndrome (SARS), four had viral pneumonia, one had pulmonary embolism, and another participant had septicemia with hypovolemic shock. Four participants died due to respiratory illnesses without a diagnosis of SARS-CoV-2 infection: diagnoses included pulmonary sepsis, acute respiratory failure, and pulmonary tuberculosis. Most infected participants who succumbed (81%) were admitted to the ICU, while 88.2% received invasive mechanical ventilation. Among participants who died with COVID (*n* = 17), 65% died after cesarean section. Comorbidities among participants who died were present in five cases (23.8%) and consisted of obesity (14.3%) or pre-eclampsia (9.5%). All 21 participants who died were unvaccinated against COVID-19.

In total, 392 of 1185 (33%) participants received at least one dose of a COVID-19 vaccine before delivery. Seventy percent of pregnant participants diagnosed with SARS CoV-2 were unvaccinated as opposed to 66% of participants in whom SARS CoV-2 was not identified, *p* = 0.09 (Table 1). Although vaccination rates were not different between infected and uninfected groups, adverse maternal outcomes (death and/ or ICU admission) were much more prevalent in unvaccinated participants, with 97% of 68 participants with an adverse maternal outcome being unvaccinated, including 93% of 44 participants with documented SARS CoV-2 in pregnancy (HR: 6.03, 95% CI: 1.45–23.7).

### 3.2. SARS-CoV-2 and Perinatal Outcomes

Among 1185 (26 twin pregnancies) participants, there were 1211 deliveries, including those of 37 stillbirths and 26 infants who died in the neonatal period. Perinatal outcomes of infants born to mothers with SARS-CoV-2 as compared to those of participants who tested negative included prematurity (30.7% vs. 23.6; *p* = 0.01), fetal distress (9.7% vs. 8.0; *p* = 0.36), stillbirths (4.7% vs. 2.6; 0.09), and neonatal deaths (3.2% vs. 1.9; *p* = 0.22), as seen in Table 2.

Among preterm births, 46% (*n* = 139) occurred due to the need to interrupt pregnancy via caesarean section because of pre-eclampsia in 33.8% (*n* = 47); SARS-COV-2 infection in 16.5% (*n* = 23); gestational hypertension in 14.4% (*n* = 20) and chronic arterial hypertension in 10.8% (*n* = 15); severe acute respiratory syndrome in 9.5% (*n* = 13); placental abruption in 5% (*n* = 7); fetal distress in 4% (*n* = 6); gestational diabetes in 1.5% (*n* = 2); HIV in 1.5% (*n* = 2); 1.5% IUGR (*n* = 2); and oligohydramnios in 1.5% (*n* = 2).

No adverse perinatal outcomes were associated with maternal SARS-CoV-2 infection according to the Kaplan–Meier analysis (Supplementary Materials Figure S1). This included all adverse perinatal outcomes (*p* = 0.20); fetal distress (*p* = 0.60); stillbirth (*p* = 0.80); neonatal death (*p* = 0.90); and prematurity (*p* = 0.20). Crude and adjusted hazard ratios of SARS-COV-2 for the time to each perinatal outcome are shown in Table 3. Even after adjusting for vaccination, comorbidity, gestational trimester, and periods, there was no difference in individual perinatal outcomes between infected and uninfected groups. In the sensitivity analysis, prematurity had an adjusted HR of 2.77 (IC 95% 1.38–5.58) (Supplementary Materials Table S3).

### 3.3. SARS-CoV-2 and Vertical Transmission

Diagnostic results (RT-PCR and serology) for SARS-CoV-2 were available for 169 of 257 exposed neonates. Vertical transmission was identified in 3.6% (6/169) of infected newborns at the time of birth. Seventy-one of 124 newborns with maternal exposure had evidence of transplacental antibody transfer with positive IgG antibodies as seen in Table 4.

Four of six newborns with laboratory evidence of SARS CoV-2 infection were admitted to the Neonatal intensive care unit (NICU) within the first hours of life due to prematurity, as shown in Table 5. All infected neonates survived.

## 4. Discussion

Building on prior evidence linking SARS-CoV-2 to adverse maternal morbidity and mortality [25], this survival analysis focuses on hospitalized pregnant participants. The risk of serious maternal outcomes within 16 days was significantly higher in those participants infected with SARS-CoV-2. An increased risk of adverse maternal outcomes in participants infected with SARS-CoV-2 infection during pregnancy in comparison to uninfected pregnant participants, even after adjusting for vaccination, comorbidity, gestational trimester, and periods of time, was observed. We observed 21 maternal deaths, 17 of which occurred in patients with COVID-19, a case-fatality rate of 6.8%, somewhat lower than the Brazilian rate of 12.7% previously reported for pregnant patients with COVID-19 [26]. We found that 24% of maternal deaths were associated with pregnancy complications and/or comorbidities, such as obesity, pre-eclampsia, and chronic high blood pressure. Obstetrical complications such as hypertensive disorders of pregnancy have been shown to be triggered by SARS CoV-2 infection in a number of studies [27,28,29]. Furthermore, studies show that the presence of comorbidities, together with advanced maternal age in SARS-CoV-2 pregnancies, also have a higher rate of complications [30,31].

Symptomatic patients usually presented with cough, fever, and shortness of breath. Patients who were symptomatic on admission had a fourfold greater risk of developing an adverse maternal outcome, corroborating previous studies [32,33,34]. In addition to greater vulnerability of pregnant and postpartum women to the development of severe COVID-19, length of symptoms from the initial phase of infection, viral load, and inflammatory responses within the pulmonary system contribute to disease progression during pregnancy [35,36,37].

The cesarean section rate was 57.0% among study participants infected with SARS-CoV-2, similar to the rates observed in Europe [38,39,40]. In our study, cesarean sections were performed due to disease severity in 58.8% of cases, with a 65% mortality rate following surgery. Nonetheless, this was expected, considering the new challenges brought about by the COVID-19 pandemic. These included limited health care access and gestational complications. Severity of illness prompted indications for delivery situations where surgical delivery may not have been shown to improve maternal and/ or perinatal outcomes [41,42,43,44]. We observed that caesarean section with SARS-CoV-2 infection was often associated with maternal death due to a higher risk of clinical deterioration and increased maternal complications in the postpartum period as already demonstrated in other studies [45,46]. Finally, severe life-limiting COVID-19 infection can be considered a non-obstetrical indication for cesarean section (C-section), similar to other rare conditions like cancer during pregnancy [47].

We found evidence of mother-to-child transmission of SARS-CoV-2 in 3.6% of cases similar to other studies [48,49]. Transplacental transmission of COVID-19 can occur due to the low levels of SARS-CoV-2 viremia and the decreased co-expression of angiotensin converting enzyme 2 (ACE2) and trans-membrane protease serine 2 (TMPRSS2) required for the entry of SARS-CoV-2 into placental cells [50]. Nevertheless, in March 2020, a study reported the first case of intrauterine transmission in the third trimester of pregnancy, with severe symptoms in the newborn, including irritability, poor feeding, axial hypertonia, and opisthotonos of the cerebral nervous system [51]. Interestingly, four of six infants who acquired SARS CoV-2 were premature, and five presented with respiratory distress requiring ICU admission. In addition, we also observed passive transfer of maternal antibodies to newborns exposed to maternal SARS-CoV-2 infection during pregnancy as reported in prior studies [52,53,54,55,56].

Adverse perinatal outcomes such as stillbirth, fetal distress, or prematurity were similar in both infected and uninfected groups after adjustment for vaccination, comorbidities, gestational trimester, and periods of time. However, we found a higher proportion of prematurity among participants who tested positive for SARS-CoV-2, as in prior research studies from the U.S. and Europe [57,58,59,60,61]. In addition, the sensitivity analysis indicated a possible association between SARS-CoV-2 infection and the risk of prematurity. Whether the preterm deliveries were iatrogenic due to performance of C-sections or other factors is not known. Further research is needed to confirm its association to COVID-19 and elucidate its mechanisms.

In low-income settings, SARS CoV-2 infection in pregnancy was associated with a high rate of adverse maternal outcomes, including death and/or ICU admission. Although the percentage of infected women having complete (two doses) vaccination was higher than that of the uninfected women, the infected PW had mild symptoms and did not experience serious adverse outcomes [25,62]. In addition, none of the participants who died were vaccinated against COVID-19. Our analysis supports previous finding of increased risk of death in unvaccinated pregnant women with pre-existing health conditions due to COVID-19 [63,64].

Only one-third of our cohort received at least one dose of the COVID-19 vaccine, which underscores the need to urgently implement preventative measures to vulnerable pregnant populations during prenatal care. To date, vaccination rates against COVID-19 in pregnancy remain low in our scenario [16,65].

Further research demonstrating the positive impact of COVID-19 vaccines in preventing adverse maternal and perinatal outcomes in low-income settings is crucial. Future study in a cohort with a larger number of PW to evaluate multiple events and dependent variables may identify additional factors that influence adverse pregnancy and perinatal outcomes.

### Strengths and Limitations

Our study’s generalizability might be limited due to recruitment at a referral hospital for higher-risk pregnancies. This could have resulted in a sample more likely to experience perinatal adverse outcomes, potentially masking any true differences between the infected and uninfected groups. However, referral hospitals are essential for capturing time-sensitive public health events like COVID-19 in pregnant women. Furthermore, the study design precluded investigation of miscarriage rates, as the majority of participants were recruited in the third trimester of pregnancy, which limited the variability of the gestational trimester throughout the study period. Another limitation is that given the study design, we cannot rule out SARS CoV-2 infection not associated with a hospital admission. As such, the number of infected participants may be an underestimate, which could potentially explain why adverse perinatal outcomes were high in both exposed and unexposed groups and were not statistically different.

Another possible shortcoming was that it was not possible to obtain the sequence of the variants or data on reinfection and asymptomatic infection during pregnancy. To address these potential limitations, we included a time period variable in the analysis intended to account for changes that occurred with the emergence of new viral variants during the pandemic. With respect to shortcomings of the study, it should also be noted that in the course of the pandemic, the participants’ vaccination status varied, reflecting changes in vaccination policies for this population that occurred during the pandemic. Our analysis incorporated a variable representing the vaccination status of each participant. However, the variable may not have captured all of the subtleties of vaccination status. Nevertheless, we attempted to address this using the time period variable, which captured the fact that vaccination was recommended for pregnant women beginning in March 2021.

## 5. Conclusions

In this cohort study spanning the first 2 years of the COVID-19 pandemic in Brazil, hospitalized pregnant women with SARS-CoV-2 infection were significantly at risk of adverse maternal outcomes. All participants who died from COVID-19 were unvaccinated. Caesarean section was frequently associated with maternal death and a high prevalence of prematurity; however, the relationship between caesarean section and maternal death in COVID-19 pregnancies has not been fully explored. Vertical SARS-CoV-2 transmission occurred in 3.6% of infants tested for the virus and was associated with prematurity and respiratory distress. Public health policies should urgently implement preventative measures against COVID-19 during pregnancy in LMIC, offering universal vaccination as a life-saving intervention.

## Figures and Tables

**Table 1:** Clinical and sociodemographic characteristics of pregnant Patients, Rio de Janeiro, Brazil, 2020–2022.

Variables	All Pregnant Women *n* = 1185	SARS-CoV-2 Infected	CI (95%) ^a^	SARS-CoV-2 Not Infected	CI (95%) ^a^	*p*-Value
		*n* = 249 (%)		*n* = 936 (%)		
Age Range						
16–19 years old	156 (13.2)	25 (10.0)	6–14	131 (14.0)	11–16	0.070
20–34 years old	867 (73.1)	181 (72.7)	66–78	686 (73.3)	70–76	
35–47 years old	162 (13.7)	43 (17.3)	12–22	119 (12.7)	10–15	
Race/ethnicity						
Other ^b^	1020 (86.1)	218 (87.5)	82–91	802 (85.7)	83–87	0.450
White	165 (13.9)	31 (12.5)	8–17	134 (14.3)	12–16	
Education						
Complete/incomplete elementary education	393 (33.1)	70 (28.1)	22–34	323 (34.5)	31–37	0.073
Complete/incomplete high school	749 (63.2)	166 (66.7)	60–72	583 (62.3)	59–65	
Complete/incomplete higher education	43 (3.7)	13 (5.2)	2–8	30 (3.2)	2–4	
Symptoms of COVID-19						
Cough	159 (41.8)	104 (41.8)	35–48	55 (5.9)	4–7	<0.001
Fever	162 (39.0)	97 (39.0)	32–45	65 (6.9)	5–8	<0.001
Dyspnea	94 (22.0)	55 (22.1)	17–27	39 (4.2)	2–5	<0.001
Myalgia	76 (22.1)	55 (22.1)	17–27	21 (2.2)	1–3	<0.001
COVID-19 Vaccination ^c^						
Complete (two doses)	209 (21.8)	54 (21.8)	16–27	155 (16.6)	14–19	0.001
Incomplete (one dose)	183 (8.5)	21 (8.5)	5–12	162 (17.3)	15–20	
Not vaccinated	792 (69.7)	173 (69.8)	63–75	619 (66.1)	62–69	
Comorbidity						
Anemia	230 (19.4)	47 (18.9)	14–24	183(19.6)	17–22	0.811
Arterial hypertension	138 (13.2)	33 (13.3)	9–18	105 (11.2)	9–13	0.374
Obesity	66 (8.4)	21 (8.4)	5–12	45 (4.8)	3–6	0.027
Asthma	52 (4.4)	16 (6.4)	3–10	36 (3.8)	2–5	0.083
Diabetes mellitus	17 (1.4)	5 (2.0)	0.7–4	12 (1.3)	0.6–2	0.375
HIV	12 (1.0)	3 (1.2)	0.3–3	9 (1.0)	0.4–1.8	0.724
Gestational Complication						
Pre-eclampsia	237 (18.5)	46 (18.5)	13–23	191 (20.4)	17–23	0.498
Gestational diabetes	93 (8.0)	20 (8.0)	4–12	73 (7.8)	6–9	0.903
Placental abruption	39 (4.0)	10 (4.0)	1–7	29 (3.1)	2–4	0.471
Prenatal consultations						
0–5 consultations (inappropriate)	483 (41.4)	103 (41.4)	35–47	380 (40.6)	37–43	0.827
≥ 6 consultations (suitable)	702 (58.6)	146 (58.6)	52–64	556 (59.4)	56–62	
Gestational Trimester						
1° trimester (≤ 13 weeks)	17 (2.0)	5 (2.0)	0.6–4	12 (1.3)	0.6–2	<0.001
2° trimester (14–27 weeks)	107 (17.3)	43 (17.3)	12–22	64 (6.8)	5–8	
3° trimester (28–42 weeks)	1061 (80.7)	201 (80.7)	75–85	860 (91.9)	89–93	
Mode of delivery (N = 1151) ^d^						
Cesarean section	526 (45.7)	135 (57.0)	50–63	391 (43.0)	39–46	<0.001
Vaginal delivery	625 (54.3)	102(43.0)	36–49	523 (57.0)	53–60	

^a^ CI confidence interval for the proportion (95%); ^b^ black or multiracial; ^c^ complete: two doses with an interval of ≥ 14 days from the RT-PCR; incomplete: first dose with an interval of ≥ 14 days from the RT-PCR and the second dose with an interval < 14 days or not vaccinated in the RT-PCR collection; not vaccinated: first dose with an interval < 14 days or not vaccinated in the RT-PCR collection; ^d^ excluded: 34 stillbirths (three twin pregnancies).

**Table 2:** Perinatal outcomes according to maternal SARS-CoV-2 infection during pregnancy, Rio de Janeiro, Brazil, 2020–2022.

Outcomes	All Pregnant Women	SARS-CoV-2 Infected ^a^	CI (95%)	SARS-CoV-2 Not Infected ^b^	CI (95%)	*p*-Value ^c^
	*N* = 1211 (%)	*N* = 257 (%)		*N* = 954 (%)		
All Adverse Perinatal ^d^						
Yes	422 (42.4)	109 (42.4%)	36–48	313 (32.8)	29–35	0.004
No	789 (57.6)	148 (57.6)	51–63	641 (67.2)	64–70	
Prematurity						
Yes	304 (30.7)	79 (30.7)	25–36	225 (23.6)	20–26	0.019
No	907 (69.3)	178 (69.3)	63–74	729 (76.4)	73–79	
Fetal distress						
Yes	101 (9.7)	25 (9.7)	6–14	76 (8.0)	6–9	0.364
No	1110 (90.3)	232 (90.3)	85–93	878 (92.0)	90–93	
Stillbirths						
Yes	37 (4.7)	12 (4.7)	2–8	25 (2.6)	1–3	0.090
No	1174 (95.3)	245 (95.3)	91–97	929 (97.4)	96–98	
Neonatal deaths						
Yes	26 (3.1)	8 (3.2)	1–6	18 (1.9)	1–2	0.229
No	1185 (96.9)	249 (96.9)	93–98	936 (98.1)	97–99	
Birth weight (g)^e^	N = 1174 (%)	N = 245 (%)		N = 929 (%)		
<2500	270 (23.0)	57 (23.3)	18–29	213 (23.0)	20–25	0.911
≥2500	904 (77.0)	188 (76.7)	70–81	716 (77.0)	74–79	

^a^ 8 twin pregnancies; ^b^ 18 twin pregnancies; ^c^ Pearson’s chi-squared test; ^d^ prematurity, fetal distress, stillbirths, and neonatal deaths; ^e^ Excluded- stillbirths.

**Table 3:** Hazard ratio of SARS-COV-2 across perinatal outcomes among 1185 (26 twin pregnancies) pregnant women from a high-risk maternity hospital in Rio de Janeiro, Brazil, 2020–2022.

Perinatal Outcome	Crude HR (CI 95%)	Adjusted HR ^a^ *(CI 95%)
All Adverse Perinatal^b^	0.96 (0.77–1.20)	0.94 (0.75–1.19)
Neonatal death	1.41 (0.63–3.14)	0.96 (0.35–2.67)
Stillbirth	1.51 (0.77–2.95)	1.07 (0.48–2.38)
Fetal distress	1.12 (0.71–1.75)	1.29 (0.82–2.05)
Prematurity (< 37 weeks)	1.05 (0.80–1.38)	0.92 (0.68–1.23)

HR, hazard ratio; CI, confidence interval. ^a^ Adjusted for vaccination status (complete vs. incomplete/unvaccinated), comorbidity, trimester, and period (up to June /2021, July /November 2021, and December/ 2021 +). *all Schoenfeld residuals were greater than 0.05: Neonatal death = 0.074, stillbirth = 0.058, fetal distress = 0.59, prematurity = 0.46. ^b^ Prematurity, fetal distress, stillbirths, and neonatal deaths.

**Table 4:** Serological and/or RT-PCR evidence for SARS-CoV-2 diagnosis among 169 in utero SARS CoV-2-exposed neonates, Rio de Janeiro, Brazil, 2020–2022.

Assay	SARS-CoV-2 Positive+ Results	IC (95%)	SARS-CoV-2 Negative Results	IC (95%)
	**N (%)**		**N (%)**	
IgG Serum (n = 124)	71 (57.2)	49–66	53 (42.8)	33–50
RT-PCR nasopharyngeal swab (n = 81)	3 (3.7%)	7–10	78 (96.2)	89–99
SARS CoV-2 IgM Serum (n = 42)	0	0	42 (100.0)	91–100
Sars CoV-2 IgA Serum (n = 90)	3 (3.0)	0.6–9	87 (97.0)	90–99

**Table 5:** Characteristics of neonatal cases of SARS-CoV-2 mother-to-child infection, Rio de Janeiro, Brazil, 2020–2022.

Case	Ballard ^a^	Weight	APGAR ^b^	Clinical signs	NICU	RT-PCR	IgA
1	34s	2265	5/6	respiratory discomfort	yes	Positive	−
2 (G1)	34s	2125	8/9	respiratory discomfort	yes	Positive	−
3 (G2)	34s	1800	4/8	respiratory discomfort; bradycardia	yes	Positive	−
4	30s	1310	6/9	sepsis	yes	Negative	Positive
5	37s	3190	4/8	respiratory discomfort	no	−	Positive
6	37s	3100	7/8	asymptomatic	no	−	Positive

^a^ Determines the newborn gestational age. ^b^ Score is a quick way for health professionals to evaluate the health of all newborns at 1 and 5 minutes after birth.

## Data Availability

The datasets used and analyzed during the current study are available from the corresponding author on reasonable request.

## Supplementary Materials

The following supporting information can be downloaded at https://www.mdpi.com/article/10.3390/v17020207/s1, Figure S1: Kaplan–Meier time from recruitment of 1185 pregnant women to severe perinatal outcome (all adverse perinatal outcomes, prematurity, fetal distress neonatal death, and stillbirth) according to SARS-CoV-2 infection status, in a high-risk maternity hospital, Rio de Janeiro, Brazil, 2020–2022; Figure S2: Multiple Cox analysis of severe maternal outcome (ICU admission or maternal death) adjusted for vaccination, comorbidity and gestational trimester, Rio de Janeiro, Brazil, 2020–2024; Table S1: Summary measures over time of outcomes in pregnant women, Rio de Janeiro, Brazil, 2020-2022; Table S2: Hazard ratio of SARS-CoV-2 infection and adverse maternal outcome (admission to ICU or maternal death), Rio de Janeiro, Brazil, 2020-2022; Table S3: Hazard ratio of SARS-CoV-2 infection and adverse perinatal (stillbirth, fetal distress, prematurity), Rio de Janeiro, Brazil, 2020-2022.
