# Supplementary material for: SARS-CoV-2 Infection and Adverse Maternal and Perinatal Outcomes: Time-to-Event Analysis of a Hospital-Based Cohort Study of Pregnant Women in Rio de Janeiro, Brazil
Source: Viruses. 2025 Jan 31;17(2):207. doi: 10.3390/v17020207 (PMC11860397; doi:10.3390/v17020207)
Supplement: Supplementary file 1 [file viruses-17-00207-s001.zip › Supplementary Table S1 (1).pdf]

Table S1. Summary measures over time of outcomes in pregnant women, Rio de Janeiro, Brazil, 2020-2022

Outcomes	Total	n events	median	0.95LCL[a]	0.95UCL[b]
**Adverse Maternal**					
Death or CTI	1,185	68	25	19	NA
**Adverse Perinatal**					
All Adverse Perinatal[c]	1,211	426	8	5	13
Prematury	1,211	304	53	20	167
Fetal distress	1211	101	NA	NA	NA
Stillbirths	1,211	37	NA	NA	NA
Neonatal deaths	1,211	26	NA	NA	NA

[a] (95% Confidence Lower Limit)

[b] (95% Confidence Upper Limit)

[c] Prematury, fetal distress, stillbirths and neonatal deaths (26 twin pregnancies)

NA (Not Available)
