# Supplementary material for: SARS-CoV-2 Infection and Adverse Maternal and Perinatal Outcomes: Time-to-Event Analysis of a Hospital-Based Cohort Study of Pregnant Women in Rio de Janeiro, Brazil
Source: Viruses. 2025 Jan 31;17(2):207. doi: 10.3390/v17020207 (PMC11860397; doi:10.3390/v17020207)
Supplement: Supplementary file 1 [file viruses-17-00207-s001.zip › Supplementary Table S2 and Table S3 (1).pdf]

Table S2. Hazard ratio of SARS-COV-2 Infection and adverse maternal outcome (admission to ICU or maternal death), Rio de Janeiro, Brazil, 2020-2022

Variables	N	Crude HR (CI 95%)	Adjusted HR[a*] (CI 95%)
Infected SARS-CoV-2	1,185	5.93 (3.58-9.84)	5.47 (3.16-9.48)
Infected SARS-CoV-2 time = 0 Excluded	1,166	6.51 (3.55-11.95)	5.29 (2.75-10.17)
Infected SARS-CoV-2 pre-eclampsia and obesy Excluded	901	5.85 (3.11-11.01)	5.69 (2.84-11.39)
Infected SARS-CoV-2 multipara Excluded	350	7.27 (2.69-19.68)	4.93 (1.69-14.81)

[a] HR, hazard ratio; CI, confidence interval. Adjusted for vaccination, comorbidity, trimester, period (up to November /2021, December /2021+), and time (1 day of follow-up, more than 1 day).

*All models meet the proportionality assumptio

Table S3. Hazard ratio of SARS-COV-2 Infection and adverse perinatal (stillbirth, fetal distress, prematurity), Rio de Janeiro, Brazil, 2020-2022

Perinatal outcome (N = 603)[a]	Crude HR (CI 95%)	Adjusted HR[b*] (CI 95%)
Neonatal death	1.49 (0.49-4.50)	-
Stillbirth	0.93 (0.29-2.93)	0.94 (0.27-3.28)
Fetal distress	0.55 (0.19-1.61)	0.58 (0.20-1.69)
Prematurity (< 37 weeks)	**2.65 (1.32-5.33)**	**2.77 (1.38-5.58)**

[a] Excluding time = 0

[b] HR, hazard ratio; CI, confidence interval. Adjusted for vaccination, comorbidity, trimester, period (up to November /2021, December /2021), and time (1 day of follow-up, more than 1 day)

It was not possible to calculate for neonatal death due to few events

*All models meet the proportionality assumptio
