# Supplementary figures and images for: SARS-CoV-2 Infection and Adverse Maternal and Perinatal Outcomes: Time-to-Event Analysis of a Hospital-Based Cohort Study of Pregnant Women in Rio de Janeiro, Brazil
Source: Viruses. 2025 Jan 31;17(2):207. doi: 10.3390/v17020207 (PMC11860397; doi:10.3390/v17020207)

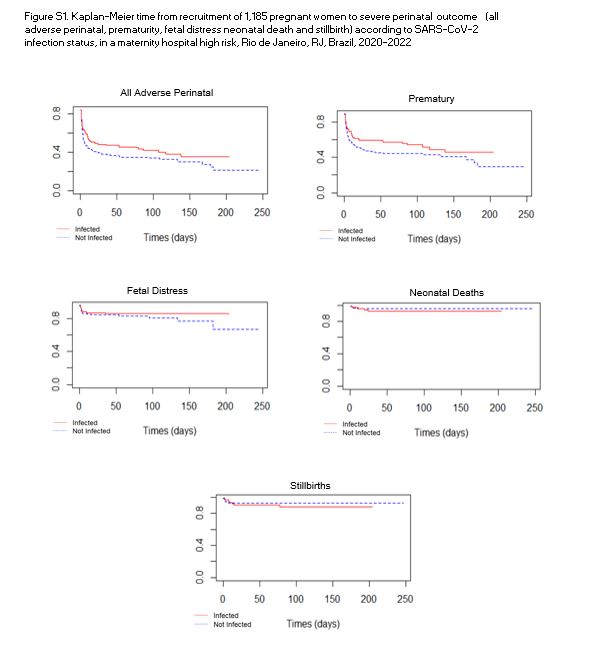

Supplement: Supplementary file 1 [file viruses-17-00207-s001.zip › supplementary Figure S1.JPG]
